# In‐Situ Monitoring the Magnetotransport Signature of Topological Transitions in a Chiral Magnet

**DOI:** 10.1002/smtd.202401875

**Published:** 2025-02-12

**Authors:** Andy Thomas, Darius Pohl, Alexander Tahn, Heike Schlörb, Sebastian Schneider, Dominik Kriegner, Sebastian Beckert, Praveen Vir, Moritz Winter, Claudia Felser, Bernd Rellinghaus

**Affiliations:** ^1^ Institute for Solid State and Materials Physics TUD University of Technology Dresden 01069 Dresden Germany; ^2^ Leibniz Institute for Solid State and Materials Research Dresden 01069 Dresden Germany; ^3^ Dresden Center for Nanoanalysis, cfaed TUD University of Technology Dresden 01069 Dresden Germany; ^4^ Institute of Physics Academy of Sciences of the Czech Republic Cukrovarnická 10 Prague 162 00 Praha 6 Czech Republic; ^5^ Max Planck Institute for Chemical Physics of Solids 01187 Dresden Germany

**Keywords:** Hall effect, in‐situ TEM, Lorentz TEM, micromagnetic simulations

## Abstract

Emerging magnetic fields related to the presence of topologically protected spin textures such as skyrmions are expected to give rise to additional, topology‐related contributions to the Hall effect. In order to doubtlessly identify this so‐called topological Hall effect, it is crucial to disentangle such contributions from the anomalous Hall effect. This necessitates a direct correlation of the transversal Hall voltage with the underlying magnetic textures. A novel measurement platform is developed that allows to acquire high‐resolution Lorentz transmission electron microscopy images of magnetic textures as a function of an external magnetic field and to *concurrently* measure the (anomalous) Hall voltage in‐situ in the microscope on one and the same specimen. This approach is used to investigate the transport signatures of the chiral soliton lattice and antiskyrmions in Mn_1.4_PtSn. Notably, the observed textures allow to fully understand the measured Hall voltage without the need of any additional contributions due to a topological Hall effect, and the field‐controlled formation and annihilation of anstiskyrmions are found to have *no* effect on the measured Hall voltage.

## Graphical abstract

The connection between magnetotransport and high‐resolution magnetic imaging is established by simultaneously monitoring magnetotransport properties and conducting in‐situ Lorentz Transmission Electron Microscopy. Ex‐situ approaches necessitated separate specimens, resulting in ambiguous correlations due to sample differences. A new direction for the field of magnetotransport is enabled, advancing its state‐of‐the‐art.

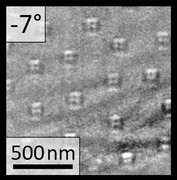

## 1 Introduction

The Hall effect in solids, discovered by Edwin H. Hall in 1879,^[^ ^1^ ^]^ describes the formation of a voltage perpendicular to the applied current and magnetic field. In materials with broken time‐reversal symmetry (e.g., ferromagnets), the Hall resistivity consists of the ordinary Hall effect (OHE) caused by the Lorentz force as well as the anomalous Hall effect (AHE) resulting from different scattering mechanisms like intrinsic, skew, and side jump scattering.^[^ ^2^ ^]^ Therefore, the measured Hall resistivity is often empirically described by

(1) $$\;\rho_{xy}=\rho_{xy}^{o}\mu_{0}H+\rho_{xy}^{a}\mu_{0}M\left(H\right)+\rho_{xy}^{t}$$

with the permeability of free space μ_0_, the external magnetic field *H* and the magnetization of the sample *M*(*H*).^[^ ^3^ ^]^ The first term describes the OHE and the second one accounts for the AHE. Even though the origin of the AHE is still subject of an ongoing debate, further contributions to the Hall resistivity, which are not proportional to the magnetization, have become of increasing research interest. Consequently, the third term designates additional contributions from the Berry phase leading to a topological Hall effect (THE). For example, the non‐vanishing Berry phase could be caused by topologically protected magnetic textures such as skyrmions and its relatives.^[^ ^4^, ^5^, ^6^, ^7^, ^8^, ^9^, ^10^, ^11^, ^12^, ^13^, ^14^, ^15^, ^16^, ^17^, ^18^, ^19^, ^20^, ^21^ ^]^ Our investigation aims to determine whether the Berry phase of topologically protected magnetic textures with finite topological charge gives rise to a measurable topological Hall effect and whether the latter can be directly related to the presence of such simultaneously observed objects.

If the THE shall be extracted from Hall measurements, the OHE and AHE have to be subtracted from the Hall signal ρ_*xy*_. The OHE is usually small and a linear subtraction is rather straightforward, but the subtraction of the large AHE requires precise knowledge of *M*(*H*) in the same specimen. If this data is not available, the interpretation of THE‐like features in magnetotransport experiments on materials with topological structures such as (anti)‐skyrmions relies on magnetometry data, micromagnetic simulations and/or magnetic imaging techniques.^[^ ^12^, ^22^, ^23^ ^]^ This has several inherent disadvantages: Micromagnetic simulations require precise knowledge of numerous material parameters, at least part of which are unknown or merely estimated. In addition, magnetometry data and/or magnetic images are usually not obtained from identical specimen, or the resolution in the magnetic images does not allow to identify the topology of the underlying textures.^[^ ^24^ ^]^ In a more sophisticated approach to extract the THE, the controversial procedure of subtracting the AHE via *M*(*H*) can be avoided by a simultaneous measurement of the Hall and Nernst effects.^[^ ^25^ ^]^ This, however, requires to also measure the (topological) Nernst effect thereby necessitating an increased experimental effort, which was not yet demonstrated for (anti)‐skyrmions.

Skyrmions and even more complex magnetic textures are frequently studied using advanced magnetic imaging techniques such as Lorentz transmission electron microscopy (LTEM) or electron holography. However, these techniques are used to identify topological textures like (anti)skyrmions in different materials as a function of magnetic field and temperature.^[^ ^23^, ^26^, ^27^, ^28^, ^29^ ^]^ Besides determining the topological charge of the magnetic texture, also dynamic studies can be performed.^[^ ^23^, ^30^, ^31^ ^]^ In transport experiments, both, magnetoresistance^[^ ^32^ ^]^ and Hall measurements^[^ ^6^ ^]^ can be performed to electrically detect skyrmions and the THE is considered to be a strong evidence for the existence of skyrmions in the material^[^ ^4^ ^]^. However, Gerber considered two independent contributions to the AHE in Equation (1) and a subtraction of only one may be erroneously misinterpreted as a THE.^[^ ^33^ ^]^

Furthermore, in magnetic systems with pronounced sensitivity to dipole‐dipole interactions, the stability of magnetic textures depends strongly on the sample geometry. This makes the correlation of magnetotransport and transmission electron microscopy (TEM) data challenging, if not conducted on the identical specimen. Fortunately, modern in‐situ TEM holders allow for the feed‐through of electrical contacts, which opens up the possibility for electrical characterization in‐situ in the microscope.^[^ ^32^, ^34^, ^35^, ^36^ ^]^ Here, we utilize our capabilities of in‐situ magnetotransport measurements in a transmission electron microscope^[^ ^37^ ^]^ to collect a set of LTEM images and corresponding Hall measurements. Using these data, we are able to look into the magnetotransport signature of antiskyrmions in Mn_1.4_PtSn.

## 2 Results and Discussion

Mn_1.4_PtSn was chosen because it is reported to host antiskyrmions at room temperature. Recent magnetotransport measurements on this material combined with magnetic imaging using the magneto‐optical Kerr effect (MOKE) by M. Winter et al. ^[^ ^24^ ^]^ had implied the occurrence of additional contributions to the Hall effect at fields just below saturation. Here, sub‐micron sized magnetic objects were observed in the MOKE images. Although the field‐induced creation of antiskyrmions was predicted in theoretical calculations, neither low‐temperature magnetic force microscopy nor the room temperature MOKE images allowed to identify the topology of these circular magnetic nano‐objects. Consequently, the origin of this additional contribution to the Hall effect and in particular the nature of the underlying magnetic texture, remained unclear. Here, LTEM provides for the necessary high resolution to clearly identify these nano‐magnetic textures.

**Figure 1:**
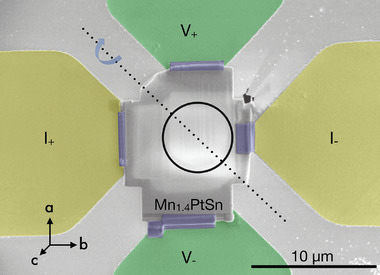
Plan view scanning electron microscopy image of the contacted Mn_1.4_PtSn lamella on a commercial Si chip with a Si_3_N_4_ window. The lamella is welded to the contact pads of the holder by local ion‐beam assisted deposition of tungsten (highlighted in purple) establishing Hall contacts for current supply I (yellow) and voltage measurement V (green) and labelled according to their polarity. A circular hole (indicated by the black circle) was cut in the free‐standing SiN membrane at the center of the chip to improve the LTEM contrast. The dashed line indicates the rotation axis of the holder in the TEM.

**Figure 2:**
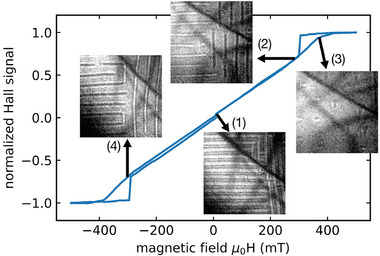
Normalized Hall signal of a thin lamella of Mn_1.4_PtSn measured in‐situ in a transmission electron microscope as a function of the magnetic field provided by the objective lens. Concurrently acquired LTEM images (1.6μm squares) depict the respective magnetic states of the sample and the arrows indicate the corresponding position on the Hall curve.

**Figure** **1** shows an overview of the prepared sample and **Figure** **2** shows the normalized Hall signal of the Mn_1.4_PtSn lamella as a function of the applied magnetic field while a current of 25μA is supplied. The selected, concurrently acquired LTEM images indicate that the observed variation of the Hall voltage is accompanied by four distinct magnetic configurations of the sample: 1) a stripe domain pattern with symmetric bright and dark contrasts in different orientational variants at low fields, 2) an increasingly asymmetric stripe pattern with field‐driven enhancement of the periodic length at intermediate fields, 3) a mixture of antiskyrmions (AS) and non‐topological bubbles (NT) and 4) the same pattern as (2) at negative fields which leads to the expected and observed inversion of the stripe contrast. Upon increasing the field, the stripe features persist up to almost saturation and vanish only at fields, where antiskyrmions and non‐topological bubbles, recognized from their typical LTEM contrast,^[^ ^28^ ^]^ occur. At roughly μ_0_ *H* = 430 mT, any magnetic contrast vanishes indicating that the out‐of‐plane magnetic saturation of the sample is reached. Upon successively decreasing the magnetic field again, this saturated state is found to persist down to a field of μ_0_ *H* = 300 mT, where the material re‐enters the previously observed asymmetric stripe phase. This re‐entrance field coincides precisely with the occurrence of a downward drop of the Hall voltage thereby correlating the hysteretic variation of the magnetic states observed in the LTEM images with the measured hysteresis in the Hall voltage between 300 mT and 400 mT. (The complete LTEM image series can be found in the extended data set,^[^ ^38^ ^]^which allows an independent re‐evaluation of our data.)

The increase of the periodic length in the stripe domain patterns is already known from chiral soliton lattices (CSL).^[^ ^39^, ^40^, ^41^ ^]^ Togawa and coworkers developed a model to describe the domain pattern of such a CSL as observed with LTEM in mono‐axial helimagnets.^[^ ^42^ ^]^ In the present work, we rather focus on the occurrence and annihilation of antiskyrmions and study their impact on the Hall effect, i.e. the field range at approximately 375 mT shown in Figure 2 configuration (3).

However, the so far considered out‐of‐plane magnetic field sweeps are not ideal to investigate the topological Hall effect. The magnetic textures observed in subsequent sweeps at fields just before reaching saturation vary from measurement to measurement: We always observe a mixture of antiskyrmions and non‐topological bubbles and their relative amount and positions vary, thereby inhibiting the disentanglement of the topological and anomalous Hall effect.

L. Peng and co‐workers have shown that by varying the in‐plane and out‐of‐plane components of the applied field through tilting the sample allows to control the relative amount antiskyrmions and bubbles, respectively, and they also reported on the material parameters to be used in the micromagnetic simulations.^[^ ^28^ ^]^ In order to systematically measure the topological Hall effect of an antiskyrmion lattice, we have therefore performed a series of tilt experiments under a constant magnetic field provided by the objective lens of the microscope. In these experiments, we were able to transform non‐topological bubbles into antiskyrmions and vice‐versa in a controlled fashion simply by adjusting the tilt angle of the sample and thereby the in‐plane field component without changing the lateral position of the object. At every tilt angle, the Hall voltage was measured and an LTEM image was acquired. Post‐acquisition alignment of the LTEM images by cross‐correlation was used to compensate for any sample drifts during tilting.

**Figure 3:**
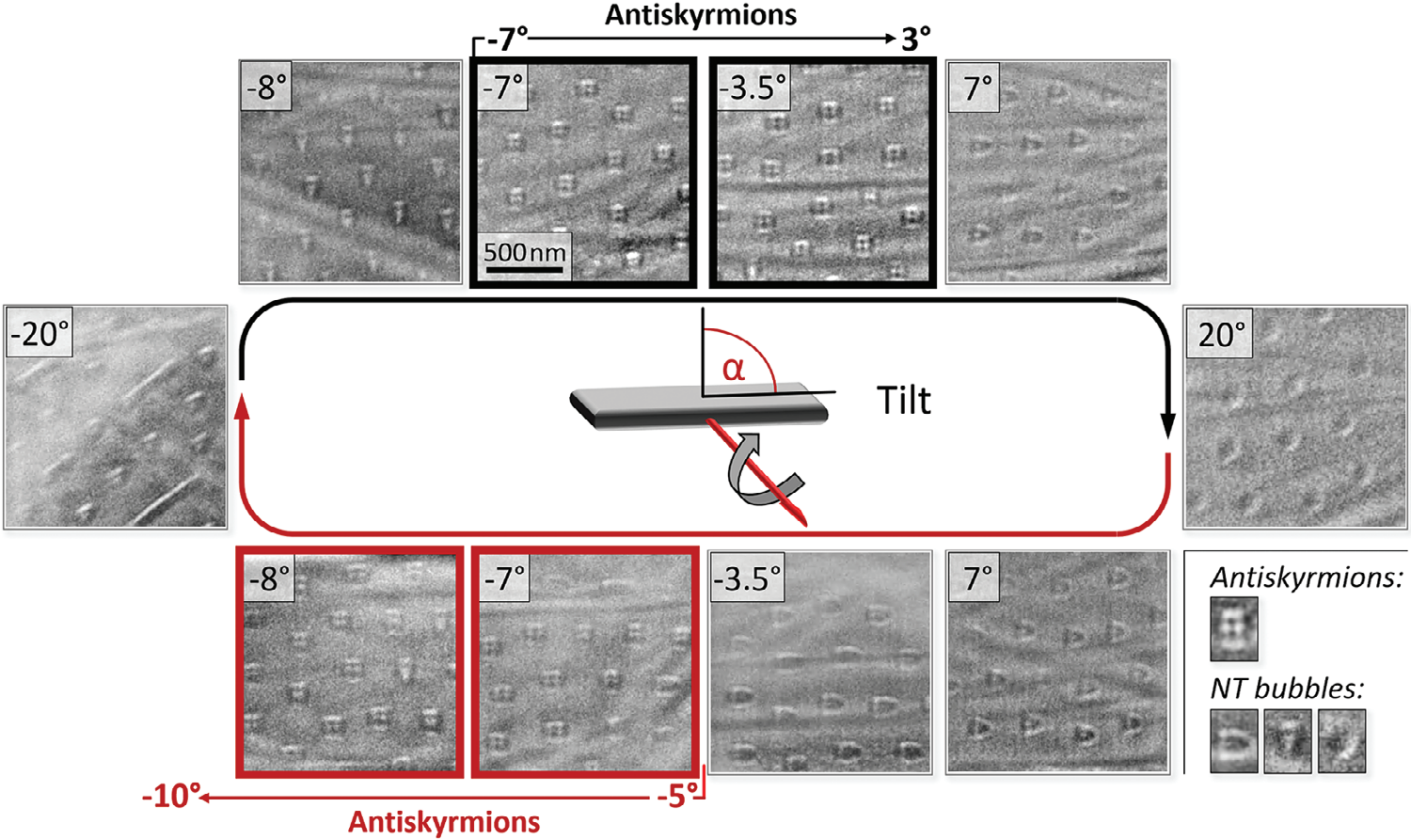
Variation of the LTEM contrast upon tilting the sample back and forth from $-7^{\circ }$ to $20^{\circ }$, $-20^{\circ }$, and back to $-7^{\circ }$. Antiskyrmions are observed to occur in the ranges $-7^{\circ }\leq \alpha \leq -3^{\circ }$ and $-5^{\circ }\geq \alpha \geq -10^{\circ }$ upon tilting forward (black) and backward (red), respectively, non‐topological (NT) bubbles occur otherwise. The differences in TEM contrast between antiskyrmions and non‐topological bubbles are indicated exemplarily in the lower right.

In **Figure** **3** we display representative examples of LTEM images acquired during this tilt series (see Figure 1 for the direction of the tilt axis and the extended data set^[^ ^38^ ^]^ for all images). The sample was initially tilted to −7° in a magnetic field of μ_0_ *H*_⊥_ = 374 mT and an electrical current of 50μA, to generate an antiskyrmion lattice. The field was chosen, since antiskyrmions were observed to occur in this range in the field sweep experiments. Upon tilting to 20° in steps of 0.5°, a non‐topological bubble lattice emerged due to the increased in‐plane field component. The sample is then step‐wise tilted back to −20°, along which it starts to form an antiskyrmion lattice at −5° and enters a mixed non‐topological bubble/helical phase. To complete the tilt loop, the sample is finally tilted back to −7°.

Non‐topological bubbles, antiskyrmions, and sections of the helical phase were manually identified, marked, and counted to determine their number from the LTEM images. However, counting the total number of antiskyrmions over the whole field of view was not possible due to the unavoidable occurrence of bending contours in the LTEM images that shifted upon tilting and inhibited the visual recognition of some of the texture elements in parts of the image. Consequently, the ratio of antiskymrions to non‐topological bubbles was used to identify those tilt angles, where antiskyrmions occur.

**Figure 4:**
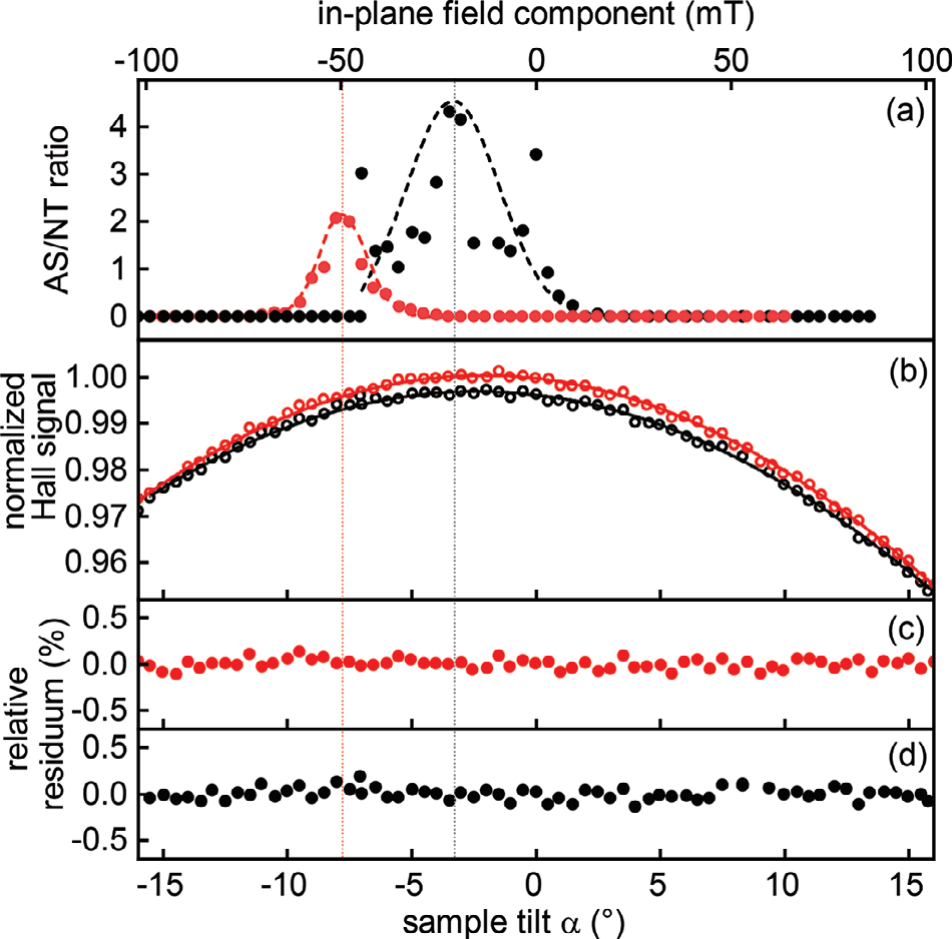
a) Ratio of antiskyrmions (AS) and non‐topological bubbles (NT) as function of the tilt angle. The resulting component of the field parallel to the plane of the sample is given on the upper abscissa. The dotted black (forward tilt) and red (backward tilt) lines indicate the angles, where the maximum number of antiskyrmions are observed in LTEM images. b) Variation of the in‐situ measured Hall signal during the same tilt series. The lines indicate cosine fits to the data with a shift of 1.5°. c,d) A subtraction of these cosine fits from the data points and subsequent normalization yields the relative residua. No peculiarities are observed upon the occurrence or vanishing of the antiskyrmions.

This ratio is plotted as a function of the tilt angle α in **Figure** **4**a, where the color‐code represents the different directions of the tilt angle (black: negative to positive angles, red: positive to negative). From this analysis, antiskyrmions are identified to occur in the angular ranges −7° ⩽ α ⩽ 3° and $-5^{\circ }\geq \alpha \geq -10^{\circ }$ during the up and down tilts, respectively, and are found to be mostly present at approximately −3.5° and −8°. Please note, that we observed up to four times more antiskyrmions than non‐topological bubbles in the sample, but never transformed all bubbles. Accordingly, the topological Hall effect is expected to be limited to roughly 80% of its expectable maximum magnitude. This slight reduction does not affect the main conclusions drawn in this manuscript.

Figure 4b shows the simultaneously measured Hall voltage normalized with respect to its maximum value as a function of the sample tilt. The Hall signal exhibits a cosinusoidal drop with increasing (positive and negative) tilt angle, which is expected assuming an originally out‐of‐plane oriented ferromagnetic background that follows the direction of the applied external field (i.e., *M*_⊥_(*H*)∝cos (α + Δα)). The slight shift of the origin of the cosine function by Δα = −1.5° is ascribed to some unavoidable mis‐tilt of the lamella imposed upon placing it onto the substrate in the in‐situ holder.

A small offset of the Hall voltage of 25 nV is found upon comparing the up and down tilts. An estimation based on the areal density of magnetic features in the LTEM images and the measured Hall voltages shows that this corresponds to exactly that amount of anomalous Hall voltage that is to be added or subtracted, when a single non‐topological bubble or antikyrmion is created or annihilated on an otherwise ferromagnetic background. We subtract the respective cosine fit for both tilt directions from the data and depict the normalized values, i.e., the relative residuum in Figure 4c,d. Most strikingly, we do *not* observe any changes or peculiarities in the course of the Hall signal that could be related to the occurrence of antiskyrmions as indicated by the AS/NT ratio in Figure 4a.

Please note that the longitudinal resistance change determined from the voltage drop across the sample between the current contacts was determined to be 0.02 % in the investigated field range and can, thus, be neglected for the interpretation of the transversal Hall voltage, while the relative error due to the limited resolution and accuracy of the measurement equipment and the reproducibility amounts to 5 nV, which corresponds to a Hall resistivity of 2.4 nΩcm. Thus, these values define the upper limit for the topological Hall effect in Mn_1.4_PtSn.

Before we conclude, let us reiterate the essential details: (i) The measured AHE is proportional to the out‐of‐plane component of the sample magnetization. (ii) Micromagnetic simulations of antiskyrmions and non‐topological bubbles show that both magnetic textures have similar out‐of‐plane components^[^ ^28^ ^]^ and, consequently, both magnetic textures have the same anomalous Hall effect. (iii) Even though completely different magnetic textures such as antiskyrmions or non‐topological bubbles are present and transformed into each other, *no impact on the Hall voltage* is observed under the conditions of our experiment. Future studies might look into the effect of the (anti‐) skyrmion size on the topological Hall effect as theoretically investigated by Matsui et al.^[^ ^43^ ^]^

## 3 Conclusion

High‐quality single crystals of Mn_1.4_PtSn were employed to investigate its magnetotransport properties, specifically the emergence and annihilation of antiskyrmions at room temperature. In situ LTEM and Hall effect measurements were conducted on micro lamellae fabricated from these crystals within an electron microscope. By following a well‐defined magnetic field protocol,^[^ ^28^ ^]^ we successfully controlled the transformation between topologically protected and non‐topological magnetic textures in the sample. Notably, antiskyrmions were first stabilized in a lattice structure, followed by their conversion into non‐topological bubbles and vice versa, without generating any Hall voltage related to these transformations. This observation contradicts expectations, suggesting that antiskyrmions do not always exhibit a (measurable) topological Hall effect. In the future, this approach can be applied to other materials hosting, e.g., chiral magnetic textures or (anti‐) skyrmions and a similar approach could be used to look into the existence of the topological Hall effect in these materials.

## 4 Experimental Section

Mn_1.4_PtSn is a non‐centrosymmetric, tetragonal compound (space group $I\bar{4}2d$) with D_2*d*_ symmetry. The lattice parameters *a* and *c* are 0.662 nm and 1.224 nm, respectively. Single crystals of Mn_1.4_PtSn were grown by the flux‐growth method using Sn flux. Please refer to Vir and coworkers for a detailed description of the single crystals growth and characterizations.^[^ ^44^, ^45^ ^]^

From one of these single crystals a TEM lamella was cut using a *FEI Helios 660* dual beam focused ion beam (FIB) system. Special care was taken to ensure a homogenous thickness of 100 nm across the lamella of approximately 8 × 14 µ in size. Commercially available SiN‐covered Si chips were used for the in‐situ electrical characterization in a *Protochips Fusion Select* holder. In order to reduce artifacts in the LTEM images, a circular hole was FIB‐cut in a freestanding SiN membrane (without any Si support underneath) at the center of the chip, over which the lamella was placed and electrically connected to the contact pads of the chip using tungsten deposition (cf. Figure 1 for details).

The in‐situ Hall measurements and LTEM investigations were conducted in a *JEOL JEM‐F200* transmission electron microscope equipped with a cold field emission gun and a *GATAN OneView* CMOS camera for fast imaging. The microscope was operated at an acceleration voltage of 200 kV. The current through the Hall bar was supplied by a *Keithley 2450* source meter, and the transverse Hall voltage was measured by a *Keithley 2182A* nanovoltmeter. The excitation of the objective lens to adjust the magnetic field exerted on the sample, the *GATAN OneView* camera used for the acquisition of LTEM images, the projective lens system of the microscope, and the electrical transport measurements are controlled by Python scripting.^[^ ^46^, ^47^, ^48^ ^]^ Please refer to our previous work for a description about the experimental equipment and the measurement procedure, including technical details of the magnetic field application and data acquisition.^[^ ^37^ ^]^

## Conflict of Interest

The authors declare no conflict of interest.

## Data Availability

The data that support the findings of this study are openly available in Opara at https://doi.org/10.25532/OPARA‐628 , reference number 628.
